# Recent Advances in Cancer Drug Discovery Through the Use of Phenotypic Reporter Systems, Connectivity Mapping, and Pooled CRISPR Screening

**DOI:** 10.3389/fphar.2022.852143

**Published:** 2022-06-20

**Authors:** Natasha Salame, Katharine Fooks, Nehme El-Hachem, Jean-Pierre Bikorimana, François E. Mercier, Moutih Rafei

**Affiliations:** ^1^ Department of Biomedical Sciences, Université de Montréal, Montreal, QC, Canada; ^2^ Lady Davis Institute for Medical Research, Montreal, QC, Canada; ^3^ Department of Medicine, McGill University, Montreal, QC, Canada; ^4^ Department of Pharmacology and Physiology, Université de Montréal, Montreal, QC, Canada; ^5^ Department of Microbiology, Infectious Diseases and Immunology, Université de Montréal, Montreal, QC, Canada; ^6^ Molecular Biology Program, Université de Montréal, Montreal, QC, Canada

**Keywords:** HTS screening, fluorescence-based assay, immunomodulatory compounds, anti-cancer therapeutics, transcriptomics, CRISPR-Cas9

## Abstract

Multi-omic approaches offer an unprecedented overview of the development, plasticity, and resistance of cancer. However, the translation from anti-cancer compounds identified *in vitro* to clinically active drugs have a notoriously low success rate. Here, we review how technical advances in cell culture, robotics, computational biology, and development of reporter systems have transformed drug discovery, enabling screening approaches tailored to clinically relevant functional readouts (e.g., bypassing drug resistance). Illustrating with selected examples of “success stories,” we describe the process of phenotype-based high-throughput drug screening to target malignant cells or the immune system. Second, we describe computational approaches that link transcriptomic profiling of cancers with existing pharmaceutical compounds to accelerate drug repurposing. Finally, we review how CRISPR-based screening can be applied for the discovery of mechanisms of drug resistance and sensitization. Overall, we explore how the complementary strengths of each of these approaches allow them to transform the paradigm of pre-clinical drug development.

## Introduction

The systematic analysis of cancer genomes has led to the identification of recurrently mutated drivers and, on a fundamental level, to a greater understanding of the diverse mechanisms of oncogenesis (Bailey et al., 2018). Clinically, this genetic characterization has further refined the classification of tumors, led to personalized treatment strategies, and guided pharmacological innovation (Liu et al., 2018). Successful examples include the development of inhibitors targeting the tyrosine kinases BCR-ABL in chronic myeloid leukemia (Druker, 2008) and ALK in a subset of lung cancers (Gristina et al., 2020).

Unfortunately, several challenges persist in the clinical translation of cancer genetic information. Importantly, several cancer alleles lead to loss of function that cannot be directly rescued by pharmacological means. In addition, the net phenotypic result of complex genetic interactions may be difficult to predict. Further, targeted inhibition of single biological pathways in malignant cells often leads to resistance that operates at several levels: outgrowth of genetically distinct subclones (Ding et al., 2012), epigenetic rewiring (Rathert et al., 2015), transcriptional heterogeneity (van Galen et al., 2019), metabolic adaptations (Stevens et al., 2020), and post-translational feedback mechanisms (Bertacchini et al., 2014). Finally, tumor extrinsic factors, such as microenvironmental cues and the immune response (Dao et al., 2021), lead to heterogeneous behavior of genetically similar cancers among patients. The identification of the functional adaptations of tumors driving resistance to therapy has led to a crucial need for potent and specific pharmacological inhibitors.

Transcending the mutational profile of cancer, screening approaches that focus on functional surrogates of biological activity, such as changes in cellular phenotype, gene, or protein expression, directly assess the link between perturbagen and desired clinical effect. Such screening strategies do not require prior understanding of the molecular target of the disease, nor of the mechanism of action of the compound. Instead, the process of phenotypic screening directly converges on biological effect. phenotypic screens can accelerate the identification of compounds targeting these adaptations, thus facilitating the bi-directional feedback between drug design and clinical observations. Complementing this approach, novel bioinformatic strategies can discover relationships between chemical compounds, molecular targets, and biological pathways, and genetic perturbation screens can identify drivers of resistance. In this review, we describe how these technological advances operate and how they have transformed cancer drug development.

## High-Throughput Screening Assays for the Discovery of Anti-Cancer Compounds

HTS assays offer the potential to accelerate the discovery and development of new pharmacological compounds for various types of medical indications (Abbott, 2003). In fact, many anti-cancer, anti-glycemic or cardiovascular drugs found on the market nowadays were initially identified via this strategy (Macarron et al., 2011). In addition, technological advancements have largely facilitated the way that HTS is conducted. For instance, large libraries can be easily and rapidly screened due to customizable robotic installations, enhanced read-out technologies as well as the ability to miniaturize assays (Liu et al., 2004).

### Biochemical and Phenotypic Assays

Biochemical assays aim to detect, quantify, or study the activity of a biological molecule in a given pathway. These would include for example, activity assays such as the colorimetric mitochondrial metabolic activity test (Mosmann, 1983), fluorescent-based assays (Titus et al., 2012; Fouda et al., 2017), receptor-binding assays (Takenaka, 2001) or disease-related assays (Ramaekers and Bosman, 2004; Zock, 2009). As the name implies, biochemical or target-based assays require prior knowledge of the desired target. Once several hits are identified using such HTS screens, the drugs’ mechanism of action are already known as they interfere with the reaction consisting of two major players (A+ B → C). This is the main strength of this type of screening and can simplify or accelerate the ability to design analogs and/or pre-clinical drug development. For example, in a sophisticated study, Z. Chen et al. (2012). designed a luminescence-based assay to screen for potential inhibitors of *Giardia lamblia* carbamate kinase, a crucial enzyme for the metabolism of this parasite. In fact, carbamate kinase converts carbamoyl phosphate into several products including ATP. The resulting ATP is then used by the luciferase luminescent enzyme to generate light. Therefore, a greater light production correlates with higher carbamate kinase’s activity. Screening almost 4,100 compounds, this assay identified enzyme inhibitors that could potentially serve as drugs against the targeted pathogen. Although biochemical screens succeed in identifying target-specific compounds, many complex biological processes induced by the drugs screened will be omitted, such as unexpected activities, toxicities or responses (Zheng et al., 2013). For instance, if the objective consists of developing an agonist molecule capable of binding a specific receptor, then an assay based on ligand-receptor binding may not be suitable as it cannot differentiate between agonist and antagonist ligands during the screening process since the effect of the ligand on signaling is not directly assessed (Zheng et al., 2013).

Despite the importance of target-based assays in drug development, phenotypic screening or ‘‘forward pharmacology’’ has contributed to the discovery of most FDA-approved drugs between 1999 and 2008, emphasizing its major role in drug discovery (Swinney and Anthony, 2011). Phenotypic screens are designed according to a disease’s characteristic, after which compounds are screened for their ability to improve the illness’s phenotype. Furthermore, prior knowledge of the drug’s mode of action is not required, while still testing its activity and efficacy. Nonetheless, the identification of the drug’s target later becomes challenging. This would also limit one’s ability to optimize the compound’s properties or develop series of analogs prior to understanding the drug’s exact mode of action. (Swinney and Anthony, 2011). Going back to the previously described example, Z. Chen et al. (2011). have also conducted a phenotypic viability assay to screen for several compounds in a HTS. The cells were treated with the compounds or control, after which the ATP levels were measured using a commercially available kit based on the luciferase activity. A greater light signal is associated with more viable cells and therefore, a less efficient drug (Chen et al., 2011). Interestingly, 28 hits in the target-based assay were inactive in the viability screen, probably because of: 1) difficulty crossing the cell membrane, or 2) the compounds were converted by the parasite’s metabolism into inactive products. This elegant example demonstrates that phenotypic screens can detect physiologically active compounds, while being more sensitive to the drugs’ pharmacokinetic properties and without knowledge of the compounds’ targets (Chen et al., 2011).

To sum up, both assays intend to provide different data related to drug discovery. As biochemical assays identify compounds hitting a specific target under study, phenotypic screens can pick-out small molecules inducing a desired change in the phenotype of the cell.

### Discovery of Compounds Targeting Identified Molecular Pathways in Cancer

While earlier versions of phenotypic HTS identified chemotherapeutic compounds through direct cytotoxic or growth-arresting effect on cancer cell lines, several of these compounds have dose-limiting toxicities on normal tissues intrinsically linked to their mechanism (such as DNA damage or inhibition of cell division). A better characterization of molecular pathways in cancer (Hanahan and Weinberg, 2000) has inspired the search for less toxic compounds specifically targeting these.

For example, in an elegant study, Sykes et al. (2016). conducted a fluorescence-based differentiation screen in acute myeloid leukemia (AML). The homeobox factor HOXA9, normally downregulated in myeloid cells, is expressed in the majority of AML, resulting in differentiation arrest (Kroon et al., 1998). An estrogen receptor-HoxA9 fusion protein was used to immortalize cultures of murine bone marrow from a reporter mouse with GFP-knocked into the lysozyme locus. Since lysozyme is a granule protein expressed in differentiated cells, the GFP expression allowed to screen for molecules capable of triggering myeloid differentiation (Faust et al., 2000). In this system, they screened 330,000 small molecules within the NIH library and identified inhibition of dihydroorotate dehydrogenase (DHODH) as a potent pro-differentiation agent. This study has sparked an interest in testing DHODH inhibitors in AML and other cancers.

Similarly, knowledge of molecular pathways that are dysregulated in cancer can inform the development of reporter cell lines in which the activity of the pathway is coupled with expression of a reporter (fluorescence or bioluminescence) amenable to HTS, a concept previously termed “mechanism-informed phenotypic drug discovery” (MIPDD) (Moffat et al., 2014). Wnt signaling is one of the key regulatory pathways of cell development and stemness and its dysregulation has been highly associated with cancer growth, particularly in colorectal cancer, but also in many more tumor entities (Zhan et al., 2017). Wnt ligands activate a β–catenin/T-cell factor (TCF)-dependent transcription program. Ewan et al. ran a cell-based assay to identify compounds that could inhibit Wnt-dependent transcription (Ewan et al., 2010). The screen used a HEK293-based reporter cell line, coding for luciferase and GFP under the control of a TCF-binding promoter. The HEK293 cells could inducibly activate Wnt through a Disheveled-estrogen receptor fusion (Dvl2-ER). As estradiol levels triggered Dvl2 activity and increased the amount of intracellular β–catenin, clones displaying TCF-dependent transcription were selected by FACS sorting for the screen. Out of the 63,040 compounds screened, 9 of those who operated at the TCF-dependent pathway were selected for further studies. While an exhaustive list of all anti-cancer drugs discovered through phenotypic screens is beyond the scope of this article, other notable examples include the use of luciferase reporters of androgen receptor or sonic hedgehog signaling to identify the inhibitor enzalutamide (Tran et al., 2009)and vismodegib (Moffat et al., 2014), respectively. An excellent review by Moffat and colleagues (Moffat et al., 2014)demonstrates that 17 of the 48 FDA cancer drugs approved between 1999 and 2013 were identified with the help of phenotypic screens.

### Discovery of Compounds Targeting the Immune System

**FIGURE 1:**
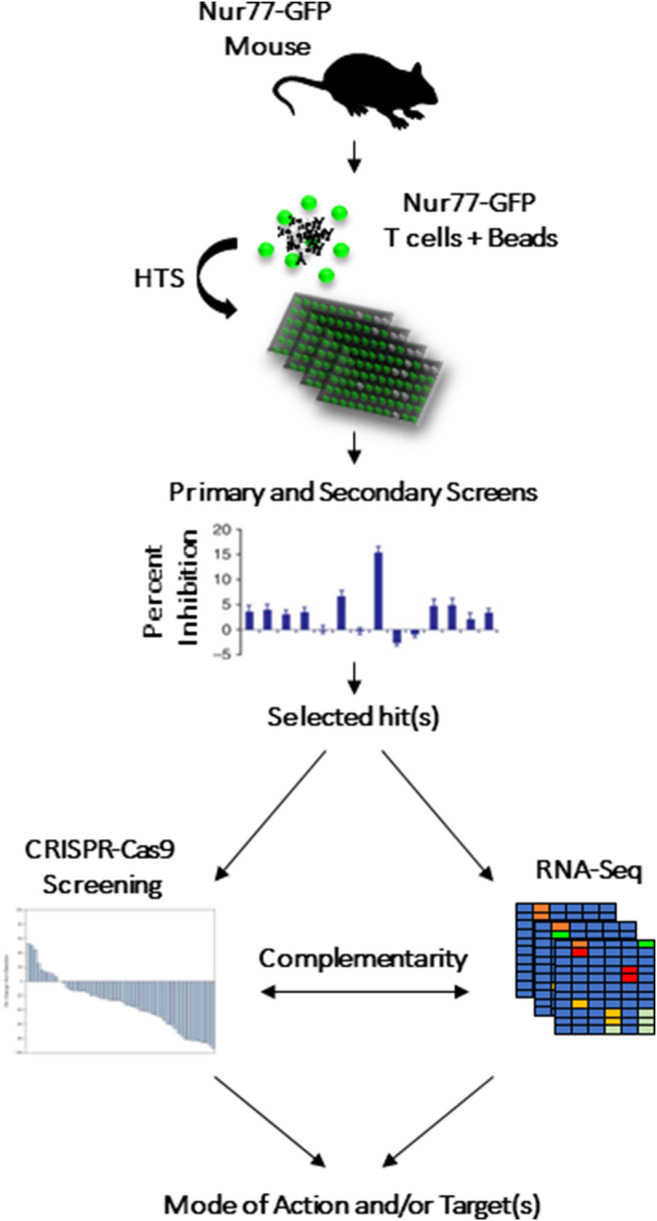
CRISPR-Cas9 and RNA-seq to complement HTS screening as means to highlight the drug’s potential target or biological pathway.

We recently described a fluorescence-based lymphocyte assay designed as a tool to screen for immunomodulatory compounds (Liu and Wang, 2012). This assay required a commercially available mouse model containing a bacterial artificial chromosome in which the Nur77 promoter is cloned upstream of the green fluorescent protein (GFP) (Fouda et al., 2017). As a result, engagement of the T-cell receptor (TCR) or the B-cell receptor (BCR) triggers the Nur77 immediate early response gene (within 3 h), which would turn on GFP expression in parallel (Ashouri and Weiss, 2017). We thus exploited this system to design a phenotypic screen centered on inhibiting T-cell activation using GFP as a surrogate marker (Figure 1) (Fouda et al., 2017). The system was then tested using a representative library containing 4,398 small molecules (chemotypes selected based on a common core structures). The primary screen led to the discovery of 160 potential hits exhibiting immunomodulatory activity. After validation, two compounds with anti-cancer properties were identified: InhiTinib and TACIMA-218. Although InhiTinib exhibited powerful suppressive activity on activated CD8 T cells, this sulfonyl-containing compound could also induce the production of reactive oxygen species (ROS) in several murine and human cancer cell lines consequently resulting in their cell death by apoptosis. Furthermore, administration of InhiTinib to mice with large, pre-established tumors significantly prolonged their survival (El-Kadiry et al., 2020). Despite differences in its molecular structure, TACIMA-218 triggered similar effects on various cancer cell lines irrespective of their p53 status, with the exception that it mainly targeted mitochondrial activity (Abusarah et al., 2021).

### Limitations of Phenotypic HTS

In 2015, Vincent et al. formulated a “rule of 3” for phenotypic screening with three criteria to assess the relevance of phenotypic assays: 1) “System” to assess how representative of the disease is the cellular assay; 2) “Stimulus” to assess how well can the experimental conditions reproduce the cellular response to study (e.g., inflammation). 3) “Readout” to assess the relationship between the assay readout and the clinical end point. From a technical standpoint, to properly identify inhibitors or activators for a given molecular target or cellular function, the quality of the assay should be first established to ensure reproducibility and the ease in quantifying its triggered signal (luminescence, fluorescence, or radioactivity). (Vincent et al., 2015).

Although phenotypic screens enabled the discovery of several first-in-class cancer drugs, they do not allow to elucidate the exact mode of action and/or molecular targets. Therefore, the use of other complementary strategies such as biochemical screening, RNA-seq and CRISPR-Cas9 screening can complement HTS findings, as they may easily narrow down the list of plausible targets.

Additionally, significant efforts are being made to account for the genetic heterogeneity found within human cancers and identify pharmaceutical compounds whose efficacy is tied to a specific genotype. In the case of RAS-mutant cancer, several comparative HTS screens could identify bioactive compounds tied to this genotype (Moffat et al., 2014). In addition, recent advances in the ability to propagate, *ex vivo*, cancer stem cells derived from patients with glioma or AML, allow to correlate genotype with drug susceptibility. The Leucegene project is an example of phenopytic HTS performed on arrays of patient-derived samples in acute myeloid leukemia (Baccelli et al., 2019).

As traditional HTS relies on the use of *in vitro* models, its efficacy in identifying drugs modulating interactions between cancer cells and their microenvironment remains limited. In fact, the tumor’s microenvironment interacts with the tumor itself, altering its response to treatments (Whiteside, 2008). Three dimensional models, or organoids, mimic better the tumor’s complexity *in vivo*. In a study by Hou et al. (2018), bioprinting-based 3D models were developed for HTS screening. This assay led to the discovery of potent anti-pancreatic cancer compounds against solid tumors, such as bortezomib, a proteasome inhibitor. Most of the drugs tested showed lower activity on the 3D models, compared to the 2D models. Yet, some showed preference in hitting the 3D model, such as disulfiram. Future HTS systems *in vitro* will likely incorporate microenvironmental interactions and multiparametric measurements of cellular response.

## Transcriptomics

A crucial strategy in drug development requires the identification of a cellular target that is functionally involved in the molecular pathogenesis of a disease (Zheng et al., 2006). Although traditional approaches are aimed to tune drugs against a single-target (enzyme, receptor, etc.) or better known as the “on-target” effect, it is now accepted that several complex pathways dictate the mechanism of action of a given drug, suggesting that “off-targets” with different biological interpretations could explain the high attrition rates when it comes to clinical trials (Waring et al., 2015). Failures due to lack of efficacy or toxicity concerns highlighted the importance of shifting towards a “one-size-does-not-fit-all” paradigm in patients’ cohorts, and consequently a routine profiling for genetic alterations is finding its way into clinical trials.

### The Era of “Big Data” and Systems Biology

In the past decade, substantial technical advances in high throughput molecular technologies, at the DNA, RNA and protein levels illuminated on the complexity of human biology and created unprecedented levels of datasets (Chen and Butte, 2016). This in turn has revolutionized another parallel discipline that combines computational methods and machine learning techniques with molecular information from multiple biological and chemical databases, to better understand the drug’s mechanism of action and link to molecular mechanisms underlying complex diseases and clinicopathological effects (Vamathevan et al., 2019).

Among the multiomics-driven molecular profiles (genomics, transcriptomics, and proteomics), genomics has enabled the first attempts to discover new druggable targets in the human genome through the analysis of genome wide association studies (GWAS), opening a new era for human genetics to inform new therapies (Visscher et al., 2017). A popular example is the “human knockout” of PCSK9 gene (proprotein convertase subtilisin/kexin type 9). Individuals with inactivating mutations in this gene are protected against coronary diseases and show very low levels of LDL cholesterol, which led to the development of PCSK9 inhibitors for the treatment of patients with genetic forms of hypercholesterolemia (Khera and Ridker, 2017). GWAS studies have been very useful in the context of cancer and have identified novel cancer-susceptibility loci. Earlier studies have focused on high penetrance genes (BRCA1/BRCA2) which warrant surveillance at the population level. A first meta-analysis of 9 GWAS studies associated 27 commonly inherited loci in 10,052 breast cancer cases to an increased risk of developing breast cancer (Michailidou et al., 2013). A recent study identified subtype-specific susceptibility loci in 133,384 breast cancer cases which informs that predisposition alleles have likely differential impact across breast cancer-subtypes and suggested novel drug targets with genetic evidence (Zhang et al., 2020).

GWAS from the United Kingdom Biobank (48,961 cancer cases) and the Kaiser Permanente Genetic Epidemiology Research on Adult Health and Aging cohorts (16,001 cancer cases) also provided insights from pan cancer studies and have detected 21 genome-wide significant associations that could identify deregulated targets and carcinogenesis mechanisms across tissue-types (Rashkin et al., 2020). United Kingdom Biobank present a unique opportunity to identify novel pharmacogenes and putative drug-targets for several complex diseases (Bycroft et al., 2018).

One of the prominent applications of GWAS is pharmacogenomics (Giacomini et al., 2012). The latter studies the relationship between genetic variations observed in thousands of individuals and corresponding drug response or metabolism. Identified genetic variations/loci (aka pharmacogenomic biomarkers) can serve as putative drug targets or risk loci when assessing drug efficacy and safety (Abbott, 2003; Karczewski et al., 2012). Bioinformatic approaches have changed the landscape of pharmacogenomic research, especially in cancer drug development (Iorio et al., 2016).

Initially, much of the pharmacogenomic (PGx) discovery was carried out in the laboratory setting through cell line resources such as the Cancer Cell Line Encyclopedia (CCLE) and the Genomics of Drug Sensitivity in Cancer (GDSC) (Barretina et al., 2012; Yang et al., 2013). PGx findings are aggregated by PharmGKB, a public interactive tool for researchers investigating how genetic variation affects drug response (Barretina et al., 2012). As described previously, genetic association studies and DNA sequencing capture the variation across the human genome and propose loss or gain-of-function hypotheses. Yet, they cannot elucidate the downstream or upstream effects underlying complex pathways. In contrast to DNA driven approaches, genome-wide transcriptional profiling (transcriptomics) can be seen as a proxy source of information for the understanding of the cellular changes under different treatments (drug vs. DMSO) or conditions (cancer vs. healthy).

Systems biology approaches using transcriptomic data from mRNA-sequencing and microarrays have been extensively used to understand perturbations caused by drug or chemical treatments to infer novel drug-targets, mechanism of action and repurposing avenues for old and existing drugs (Barretina et al., 2012). Public transcriptome databases can be leveraged to inform target selection and validation. Functional hypotheses can be interrogated from high throughput experiments such as mRNA expression in normal tissues (GTEX), cancer patients from The Cancer Genome Atlas (TCGA), or databases spanning a wide range of diseases, model organisms and multiple tissue types, such as the Gene Expression Omnibus (GEO) repository and the ArrayExpress Archive of Functional Genomics Data (Barrett et al., 2013; Cancer Genome Atlas Research Network et al., 2013; GTEx Consortium, 2015). More recently, machine learning and deep learning algorithms have used CRISPR-Cas9 genetic perturbation and transcriptomic data from thousands of cancer cell lines to predict biomarkers of drug response and cancer dependencies.

The following section will discuss the use of transcriptomics to connect or cluster similar drugs, infer novel drug-target predictions and identify novel drugs that could reverse molecular states (aka drug repositioning) as a cost-effective alternative to the classical drug discovery pipeline.

### Drug-Induced Transcriptomics Provide Insights Into the Changes Within Molecular Pathways and a Better Understanding of Drugs’ MoA

In 2006, the connectivity map (CMap) pioneered the concept that commonalities in drug mechanism of action can be inferred from similar transcriptional responses upon treating cancer cells with 1,309 bioactive compounds (Lamb et al., 2006). It was superseded by the LINCS dataset expanding to over 1 million gene expression profiles spanning ∼20,000 chemical perturbations. The idea can be summarized as follows: 1) Identifying a new chemical/drug of interest with unknown property or MoA; 2) Experimental design consisting of drug-treated cells vs. untreated or vehicle controls (can be *in vitro* and *in vivo*); 3) Extraction of mRNA and capturing global molecular perturbations via RNA-seq or microarray assays; 4) Applying bioinformatic methods (e.g, differential gene expression analysis) to extract a robust drug induced gene expression signature that discriminate treated groups at different dose levels and time points; 5) Applying statistical algorithms (e.g, connectivity mapping) to score up and down-regulated genes with respect to similar expression patterns induced by reference compounds in the connectivity map.

This “guilt by association” concept generates new hypotheses for uncharacterized compounds. This approach has been applied successfully to understand the MoA of celastrol, a natural herbal compound (Engerström, 1990). Celastrol’s gene expression mimicked HSP90 inhibitors and its activity on reducing the androgen receptor-HSP90 interaction has been experimentally validated in the LNCaP prostate cancer cell line (Hieronymus et al., 2006). Although celastrol shared a low chemical similarity with these HSP90 inhibitors, it was intriguing how similarity in transcriptional profiles can be independent of chemical structure (Chen et al., 2015).

Given that structural and molecular layers are complementary, several recent studies have used integrative computational approaches to elucidate drug MoA and propose new repurposing avenues. A versatile method known as Drug Network Fusion (DNF), has fused drug-centric networks relying only on basic drug characteristics such as structural information from pubchem and NCI60, and drug-induced perturbation profiles from LINCS and CMap (Shoemaker, 2006; Subramanian et al., 2017; Kim et al., 2019). DNF taxonomy identified most of the known drug communities, was able to capture on and off-target effects and demonstrated the scalability of unsupervised network methods in the context of drug repurposing (Koleti et al., 2018).

### Applications of Drugs- and shRNA-Induced Transcriptomics to Identify Potential Drug-Target Interactions

The LINCS database contains both gene expression profiles of ∼20,000 chemical treatments and ∼13,000 shRNAs targeting 3,800 genes across 9 cell lines (El-Hachem et al., 2017a; Subramanian et al., 2017; Koleti et al., 2018) . This setting can be used to infer novel drug-target interactions by correlating expression profiles from a specific gene knock down (KD) with drug-induced expression profiles. Pabon et al. (2018). have trained a random forest classifier (RF) on a set of 29 FDA drugs and found that this machine learning algorithm correctly identified the target in the top 100 for 16 out of 29 FDA approved drugs (55%). They also found that for some compounds such as proteasome inhibitors, chemical-induced perturbations correlated well with the corresponding gene KD (e.g, PSMA1). From a target-centric perspective, and using the RF predictive algorithm, they identified phloretin and RS-39604 as potential inhibitors of HRAS/KRAS respectively and validated their activity at μM concentrations. They further showed that wortmannin, a PI3K inhibitor, binds PDK1. This has been validated by a model of wortmannin bound to the PDK1 catalytic domain and assessed with a PDK1-PIP3 interaction-displacement assay which resulted in a decreased PIP3 interaction with increasing concentrations of wortmannin. In contrast to other classical methods that rely on chemical similarity, methods based on drug-induced molecular perturbations provide an unprecedented opportunity for polypharmacological therapies and repurposing by identifying key pathological pathways shared by multiple disease modules and prioritizing drug targets that can lead to novel therapeutic alternatives (El-Hachem et al., 2017b; Peyvandipour et al., 2018; Chan et al., 2019).

### Applications of Drug-Induced Transcriptomics for Drug Repurposing

Since the creation of the earlier version of the connectivity map (CMap), drugs that induce an anticorrelated expression profile with respect to a given disease expression signature could be considered as therapeutic agents. Using this algorithmic technique, it was hypothesized that in a given “disease state,” a set of perturbed gene sets or biological pathways can return to a baseline or “normal state” following an effective drug treatment. Sirota et al. (2011), showed that the antiulcer drug cimetidine reduced tumor formation in lung adenocarcinoma using mouse xenograft models. Chen et al. (2017a). identified anthelmintic drugs as potential therapeutic candidates in hepatocellular carcinoma (HCC). The disease signature was built by contrasting gene expression profiles from cancer patients and normal liver tissue from TCGA (Cancer Genome Atlas Research Network et al., 2013). Among the tested FDA-approved drugs, niclosamide showed strong reversal of the gene expression signature in HCC and its efficacy was confirmed *in vitro*, in patient derived xenografts and in genetic engineered models of HCC. Interestingly, the measured reversal of expression by a compound correlates with its efficacy in the tested cell lines. This systems pharmacology approach is gaining popularity for precision medicine applications (Chen et al., 2017b).

Gene expression (microarray and RNA-sequencing) has been widely harnessed to understand drug-induced transcriptional perturbations and how it implicates drug repurposing, target and biomarker identification. Still, CRISPR/Cas9 functional genomic assays are now powerful tools to identify context dependent drug-targets; its combination with transcriptomics could offer a path forward in drug discovery and development.

## Genetic Perturbation Screens

The most common approaches to study the causal link between genes and cellular responses involve perturbing normal gene expression using techniques that either: 1) alter the DNA sequence of a gene to inactivate it (e.g., Cas9 or Cas12); 2) alter levels of expression (inactive Cas9 fused with transcriptional activators or repressors, or 3) repress a gene by targeting mRNA transcripts for degradation (shRNAs). The advantages of both CRISPR and shRNA screening technologies are their scalability and flexibility. Using simple rules for the design of gene targeting sequences, most protein-coding genes in the genome can be efficiently perturbed. Thousands of shRNA and CRISPR screens have been reported at the scale of the genome in various contexts, ranging from identifying genes that are essential in malignant cells, to those that mediate resistance to chemotherapy or promote immune responses *in vivo* (Zuber et al., 2011; Tzelepis et al., 2016; Manguso et al., 2017; Wang et al., 2017; Dong et al., 2019).

In their simplest form, genetic perturbation screens can be performed to identify genes that are essential for cellular growth (Aguirre et al., 2016; Munoz et al., 2016; Tsherniak et al., 2017). The Cancer Dependency Map (https://depmap.org/portal/) is a collaboration between the Broad Institute and the Wellcome Sanger institute (Aguirre et al., 2016; Munoz et al., 2016), which screened the genome of more than a thousand cancer cell lines. The screening of many cancer cell lines from different tissues has enabled the identification of genes that are specifically essential depending on the tissue of origin or mutated oncogenic driver. For example, comparisons of screening results between RAS-mutated and RAS-wild-type leukemic cells identified essential partners of oncogenic RAS(75).

### Pharmacogenomic Screens

Since pharmaceutical compounds elicit a cellular response characterized by coordinated gene expression programs, genetic perturbation screens performed in the presence of compounds can identify which genes are essential to such responses (Colic and Hart, 2019).

CRISPR-based pharmacogenomic screens are widely used to help identify drug targets and characterize mechanisms of therapeutic resistance or sensitivity. In recent years, genome-wide CRISPR-Cas9 screens have successfully been used to identify candidate targets across diverse cell types. In 2017, a screen by Hou et al. found that loss of the genes SPRY3 and GSK3 drives resistance to FLT3-inhibition in acute myeloid leukemia (AML) (Hou et al., 2017). FLT3-inhibitors are currently in clinical trials as a monotherapy and in combination with chemotherapy for the treatment of AML. However, many patients ultimately develop resistance. Hou et al. (2017). further demonstrated that SPRY3 and GSK3 expression correlates with clinical resistance to the FLT3-inhibitor Quizartinib in primary human AML samples, and inhibition of their downstream pathways re-sensitizes AML cells to Quizartinib *in vitro*. In addition to providing a novel resistance mechanism to FLT3-inhibition in AML, these hits offered a strategy for targeting of new pathways to lessen resistance.

Dr. Daniel Durocher’s group in Toronto have similarly used genome wide CRISPR screening to study genes driving sensitivity to PARP inhibition in human cancer cell lines. In 2018, Zimmermann et al. (2018). published a series of CRISPR screens demonstrating that loss of the genes coding for RNase H2, an enzyme complex not previously linked to response to PARP-inhibition, is synergistically lethal with PARP-inhibitors (Zimmermann et al., 2018). The authors performed an initial set of CRISPR screens to identify the RNASEH2 genes as powerful sensitizers to PARP-inhibition, followed by a second set of screens in RNase H2-KO cell lines to examine the mechanisms underlying this result. This work demonstrated that deficient RNase H2 activity impairs ribonucleotide excision repair, producing PARP-trapping lesions and causing genomic damage.

### Experimental Considerations

**FIGURE 2:**
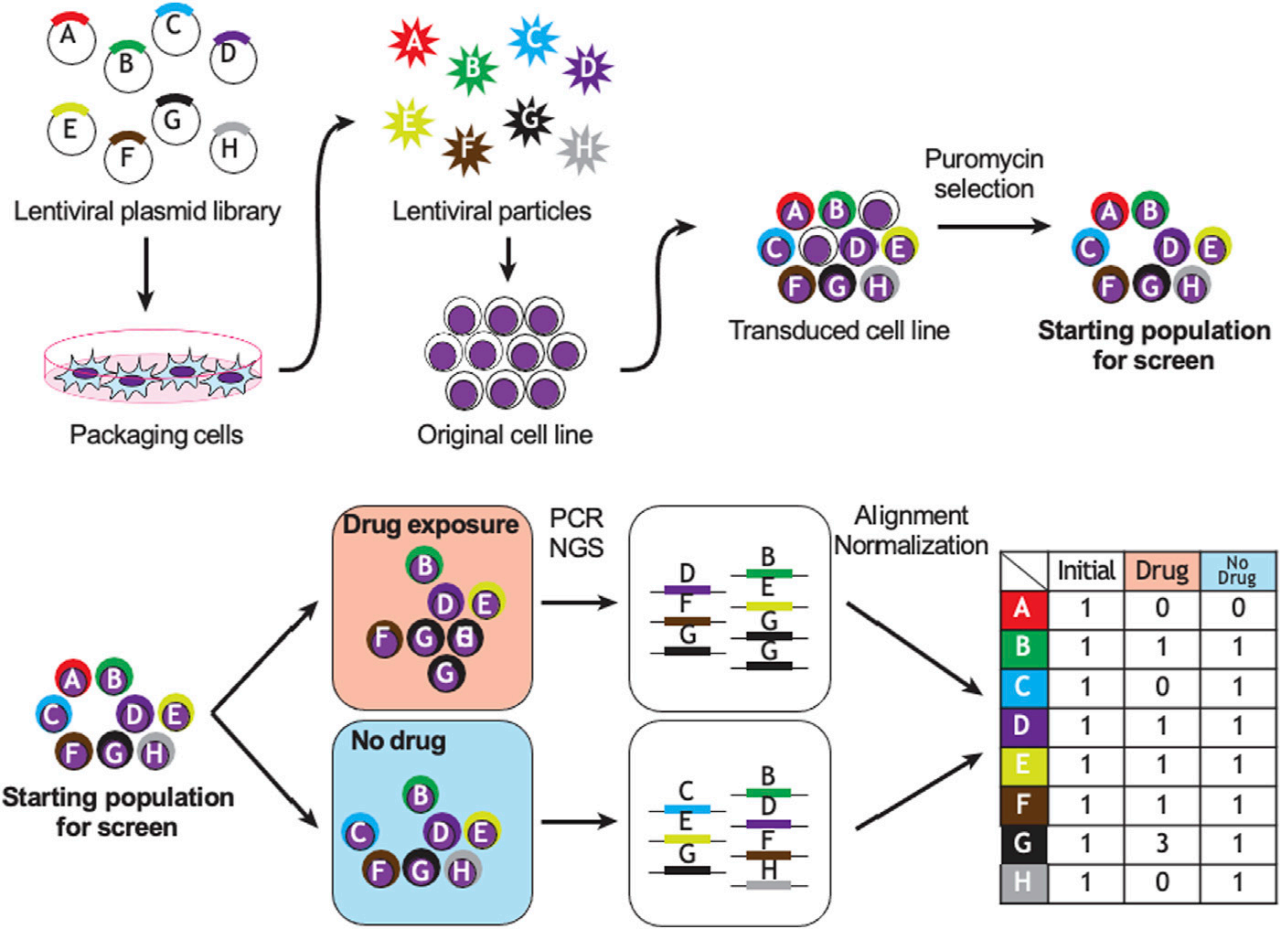
Example of CRISPR or shRNA pharmacogenomic screen. Sequence A targets a gene essential to cell survival; B, D, E, F are biologically neutral; G promotes drug resistance and C and H promote drug sensitivity. PCR: polymerase chain reaction, NGS: next-generation sequencing.

Figure 2 demonstrates a typical workflow for CRISPR pharmacogenomic screening. Pooled genetic screens involve transducing cells with a pool of diverse shRNA or sgRNA sequences, targeting a group of genes simultaneously, then measuring their relative abundances over time using next-generation sequencing. When shRNA or sgRNA sequences are delivered within the cells using lentiviruses, the sequences integrate in the genome and are propagated to daughter cells. By counting the abundance of these sequences over time in the population, it is possible to infer their biological effect in various conditions, based on a fixed stoichiometry between shRNA or CRISPR sequences and dividing cells due to lentiviral integration. The optimal experimental design of a pooled genetic perturbation screen aims to minimize stochastic changes in sgRNA abundance due to cell culture or passaging, while maximizing the statistical power to detect biologically meaningful effects. Some important experimental concepts are defined in Table 1.

**TABLE 1:** Frequently used concepts in CRISPR screening.

Concept	Definition
Coverage	Average number of cells infected by each sgRNA or shRNA. Calculated by dividing the total number of cells by the total number of sgRNA or shRNA in the library. Example: “cells were propagated at a minimum coverage of 200x”
Sequencing depth	The number of next-generation sequencing (NGS) reads mapped to sgRNA or shRNA sequences
Recovery	The fraction of the library for which a sgRNA or shRNA is detected in the NGS data x number of times. Example: “80% of the library was recovered at least 5 times”
Log2 fold-change (Log2FC)	Change in abundance of individual sgRNA- or shRNA-infected cells, normalized for sequencing depth, between two conditions, Log2-transformed. Example: Log2FC of 3 = 8-fold increase in abundance
Z-score	Number of standard deviations below or above the mean of a given Log2FC in comparison to the distribution of all Log2FC values. It is often used to report the effect of perturbing individual genes by integrating the Log2FC values for all sequences targeting the gene
Dropout	Loss of representation of a sgRNA or shRNA among the library recovered by NGS, either due to a biological effect or stochasticity
Bottleneck effect	Random dropout of sgRNA- or shRNA- infected cells from a population due to sampling of a small number of cells, for example during passaging with high dilution

Methods for CRISPR genetic screening have rapidly improved and new tools and applications are continuously published. Using data from previous genome-wide CRISPR screens, linear regression models and deep learning algorithms have been produced to improve the design of sgRNAs and reduce the potential for off-target effects (Wang D. et al., 2019). Online tools with these models are now available to help predict the on-target activity of the user’s sgRNAs and select sgRNAs for Cas9 enzymes binding a variety of PAM sequences. The selection of sgRNAs with fewer off-target effects considerably improves the coverage of sgRNA libraries and reduces the potential of false positive results. Several validated human genome-wide libraries, such as the GeCKO v2 (Sanjana et al., 2014), Toronto KnockOut (Hart et al., 2017), and Broad Brunello libraries (Doench et al., 2016), have been made publicly available to increase the accessibility of CRISPR screening.

From the standpoint of genetic perturbation screening, it is important to consider the different effects of CRISPR and shRNA on gene expression. CRISPR often leads to bi-allelic gene knockout through the introduction of small insertions or deletion. Consequently, if a given gene, involved in the response of a cell to a pharmaceutical compound, is also essential to survival of the cell, it will likely not be identifiable in a pharmacogenomic CRISPR screen. In contrast, shRNAs have a partial and varied effect on mRNA transcript abundance, so they might be better suited for the study of the subset of genes that are essential to survival of the cell. However, a significant drawback for shRNA screening is the greater occurrence of “off-target” effects with shRNA: the targeting sequence of shRNAs may affect other non-intended mRNA transcripts, thus affecting the interpretation of some experimental results. To circumvent this issue, the CRISPR system can be repurposed to transiently enhance or repress gene expression instead of producing a gene knockout (Maeder et al., 2013; Qi et al., 2013). The CRISPR activation (CRISPRa) system involves the fusion of deactivated Cas9 to a transcriptional activator domain. The deactivated Cas9 no longer cleaves the DNA; instead, the activator domain recruits the transcriptional machinery to enhance expression of the target gene (Maeder et al., 2013). Similarly, CRISPR interference (CRISPRi) is comprised of inactive Cas9 fused to a repressor domain which acts to temporarily reduce gene expression (Qi et al., 2013). In some cases, CRISPRi may be preferable to the traditional CRISPR system which can generate multiple DNA breaks and induce a DNA damage response (Haapaniemi et al., 2018).

### Computational Pipelines

A variety of publicly available resources exist for the analysis of CRISPR screening data. The complete analysis of CRISPR-Cas9 screens requires multiple steps after NGS, including data normalization, quality control, and identification of positively or negatively selected genes and relevant biological pathways (Li et al., 2014). Some of the most commonly used computational tools for CRISPR data analysis are the MAGeCK, BAGEL, CERES, and drugZ algorithms. These methods are based on different statistical approaches and are each suited to specific experimental designs.

The MAGeCK (Model-based Analysis of Genome-wide CRISPR-Cas9 Knockout) algorithm was published in 2014 and is one of the most popular comprehensive methods for analyzing CRISPR data. MAGeCK uses a negative binomial model to test whether sgRNAs differ significantly between conditions and produces a list of FDR-adjusted hits (Li et al., 2014). Two updated versions of MAGeCK, MAGeCK-VISPR and MAGeCK-Flute, were released in 2015 and 2019 by the same group (Li et al., 2015; Wang B. et al., 2019). The MAGeCK-Flute package adopts the MAGeCK version suitable for the selected experimental design and offers pathway enrichment analysis and data visualization functions (Wang B. et al., 2019).

The BAGEL (Bayesian Analysis of Gene EssentiaLity) pipeline was developed to leverage data from previous CRISPR screens. BAGEL uses essential and non-essential reference gene sets to identify novel essential genes in a screen and provides a Bayes factor for each gene (Hart and Moffat, 2016). Although the BAGEL algorithm is highly sensitive, the reference gene list requirement limits its use.

Many cancer cell lines show high copy number variation (CNVs). In CRISPR-cas9 screening, this presents an issue as sgRNA targets within high CNV regions may cause multiple DNA double-stranded breaks and cell death independent of gene essentiality (Meyers et al., 2017). CERES was designed to compensate for this phenomenon by normalizing changes specific to a cancer cell line. This reduces the number of false positives; however, the algorithm requires CNV profiles from multiple cell lines which may not be available for all screens (Meyers et al., 2017).

The drugZ algorithm was developed specifically for the analysis of pharmacogenomic CRISPR screens. DrugZ calculates gene-level normalized Z-scores and FDR values, and can be applied to identify genes involved in conferring drug resistance and sensitivity (Colic et al., 2019).

Though widely used, a shortcoming of the described packages is the required programming knowledge. More recently tools such as Cas-analyzer and PinAPL-Py have been published to provide complete web-based CRISPR analysis pipelines for researchers with less computational experience (Park et al., 2017; Spahn et al., 2017).

### *In Vivo* Screens

Although the majority of CRISPR-Cas9 screens are performed *in vitro*, the technology has also been adapted for *in vivo* use (Chow and Chen, 2018). *In vivo* screening provides a better disease model in contrast to *in vitro* cultures and more accurately recapitulates the microenvironment of the chosen cell type. Advanced forms of *in vivo* screening involve the use of Cas9 transgenic animals. In these screens, the sgRNAs are intravenously injected or directly administered to the target site within the animal and act directly within the chosen tissue (Chow and Chen, 2018). In immunocompetent animals, this system also mimics the immune involvement of the target site. However, this method has the significant challenge of achieving the desired number of transfected cells, sgRNA coverage and MOI within the chosen tissue which may be poorly accessible (Chow and Chen, 2018). Another method of *in vivo* screening is the use of indirect or transplant screens, which are performed by transplanting knockout cells generated *in vitro*. Transplant screens similarly involve the target site microenvironment; however, the engraftment rates of the transplanted cells are highly variable, and some models may require large numbers of cells to be successful. Indirect screens also often require immune deficient hosts which less faithfully reflects the tissue microenvironment and disease pathogenesis (Chow and Chen, 2018).

## Conclusion

The accumulated knowledge gained on disease pathophysiology combined to the urgent need for new small molecules with outstanding therapeutic potential highlight the importance and potential of HTS. Although biochemical-based HTS allow the discovery of chemotypes with known mode of action or target, phenotypic screening provides a list of compounds displaying a desired biological effect in the absence of target(s) knowledge. Besides, the identification of genes or phenotypes through pharmacogenomic or phenotypic screens do not always provide information concerning the pharmacological properties of the identified/lead compound. Thus, phenotypic screening requires almost always complementary biochemical approaches to fully characterize the molecular target binding and MoA of the compound of interest. A possible combinatory strategy or alternative would consist of designing a library of competitive antagonists or negative allosteric modulators prior to their screening in a phenotypic assay. The latter approach is particularly interesting as negative allosteric modulators are usually known for their higher target selectivity compared to competitive antagonists. This would then result in better selectivity and less side effects while exhibiting the desired biological effect (Ni et al., 2019; Slosky et al., 2021). Simultaneously, advancements in the field of systems biology have led to the development of various strategies capable of linking chemotypes with modulated target(s), whereas CRISPR and shRNA pharmacogenomic screens can identify genes essential to the pharmacological property of the compound. CRISPR and shRNA screens performed in the context of drug exposure are especially useful to identify biological pathways in cellulo that modulate sensitivity or resistance, which can inform predictive models of patient response (when gene expression data is available) or the development of pharmacological synergies (when “druggable” targets are identified).These powerful and synergistic approaches have led to the approval of several new drugs and herald a bright future for cancer drug development (Moffat et al., 2014).

## Glossary

AML: acute myeloid leukemia
BAGEL: bayesian analysis of gene essentiality
BCR: B-cell receptor
CCLE: cancer cell encyclopedia
CMap: connectivity map
CNV: copy number variation
CRISPR: clustered regularly interspaced short palindromic repeats
CRISPRa: CRISPR activation
CRISPRi: CRISPR interference
DNF: drug network fusion
GDSC: genomics of drug sensitivity in cancer
GEO: gene expression omnibus
GFP: green fluorescent protein
GWAS: genome wide association studies
HCC: hepatocellular carcinoma
HTS: high-throughput screening
KD: knock down
LDL: low-density lipoproteins
MAGeCK: model-based analysis of genome-wide CRISPR-Cas9
MIPDD: mechanism-informed phenotypic drug discovery
MOI: multiplicity of infection
NGS: next generation sequencing
PAM: protospacer adjacent motif
PCR: polymerase chain reaction
PCSK9: proprotein convertase subtilisin/kexin type 9
PGx: pharmacogenomics
RF: random forest classifier
ROS: reactive oxygen species
sgRNA: single guide RNA
TCF: β–catenin/T-cell factor
TCGA: the cancer genome atlas
TCR: T-cell receptor
UKB: United Kingdom biobank
